# Supplementary figures and images for: Full-Length Transcriptomes and Sex-Based Differentially Expressed Genes in the Brain and Ganglia of Giant River Prawn Macrobrachium rosenbergii
Source: Biomolecules. 2023 Mar 2;13(3):460. doi: 10.3390/biom13030460 (PMC10046887; doi:10.3390/biom13030460)

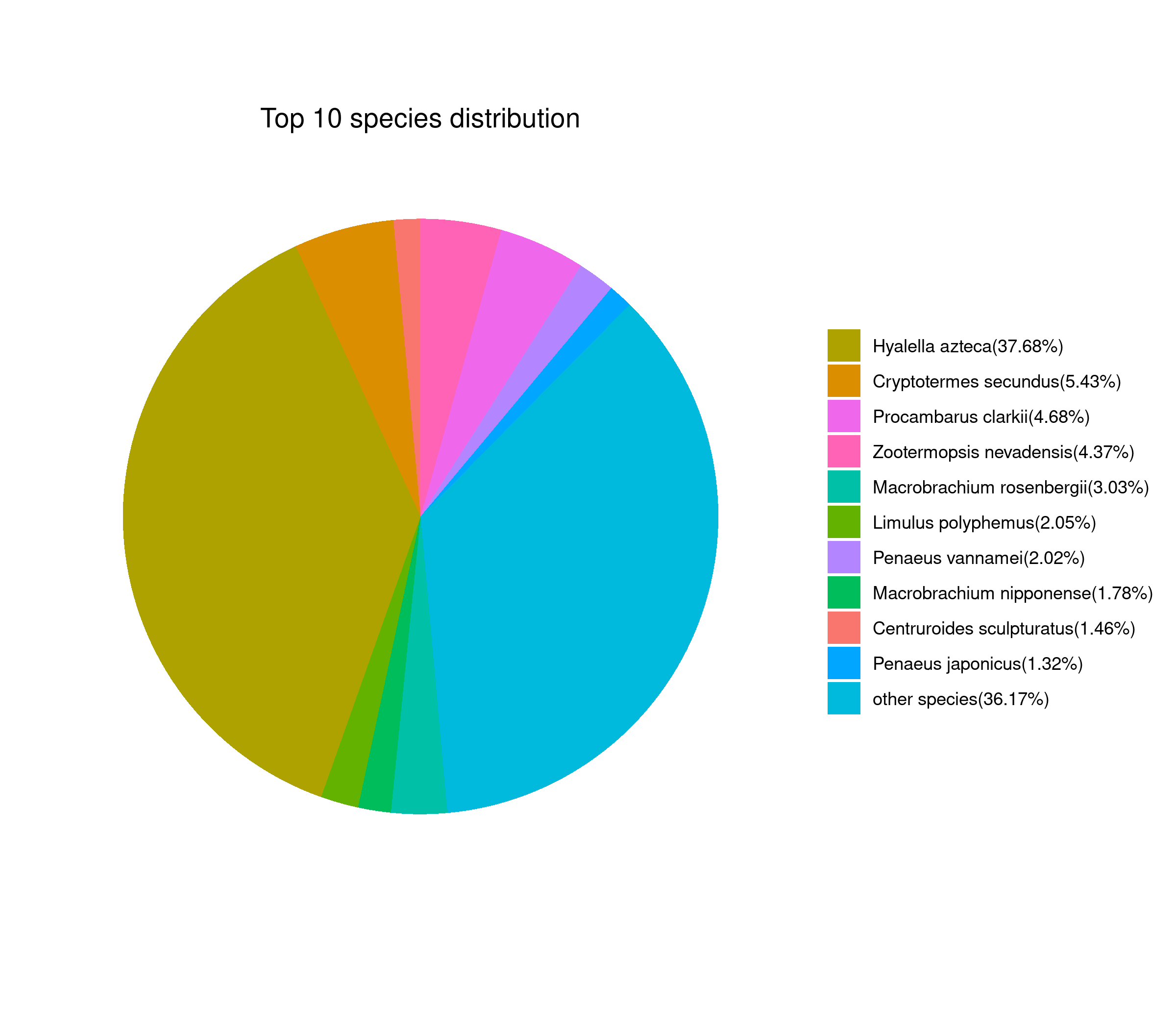

Supplement: Supplementary file 1 [file biomolecules-13-00460-s001.zip › Figure S1 Unigene.NR.species.top10.png]

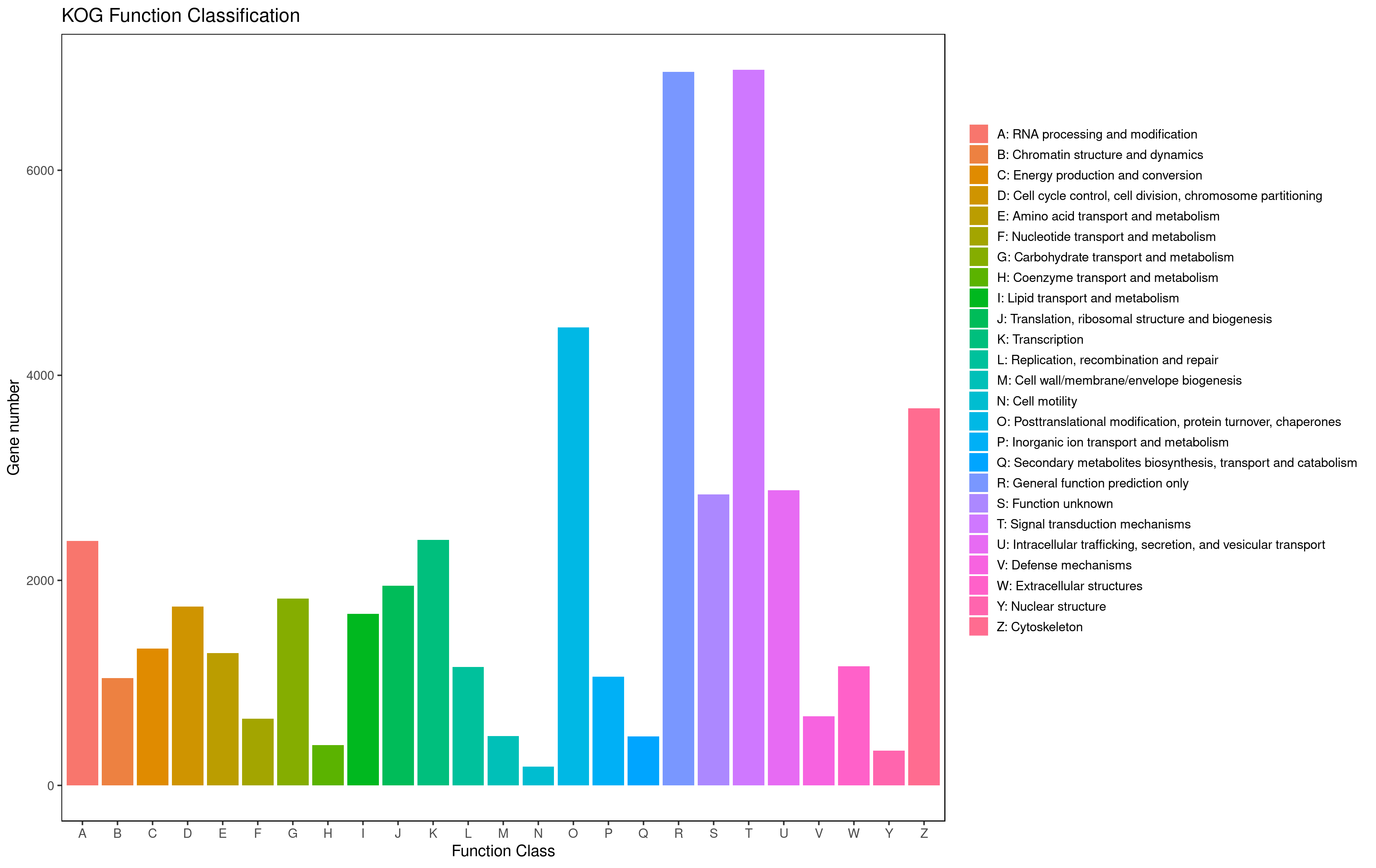

Supplement: Supplementary file 1 [file biomolecules-13-00460-s001.zip › Figure S2 Unigene.KOG.tif]

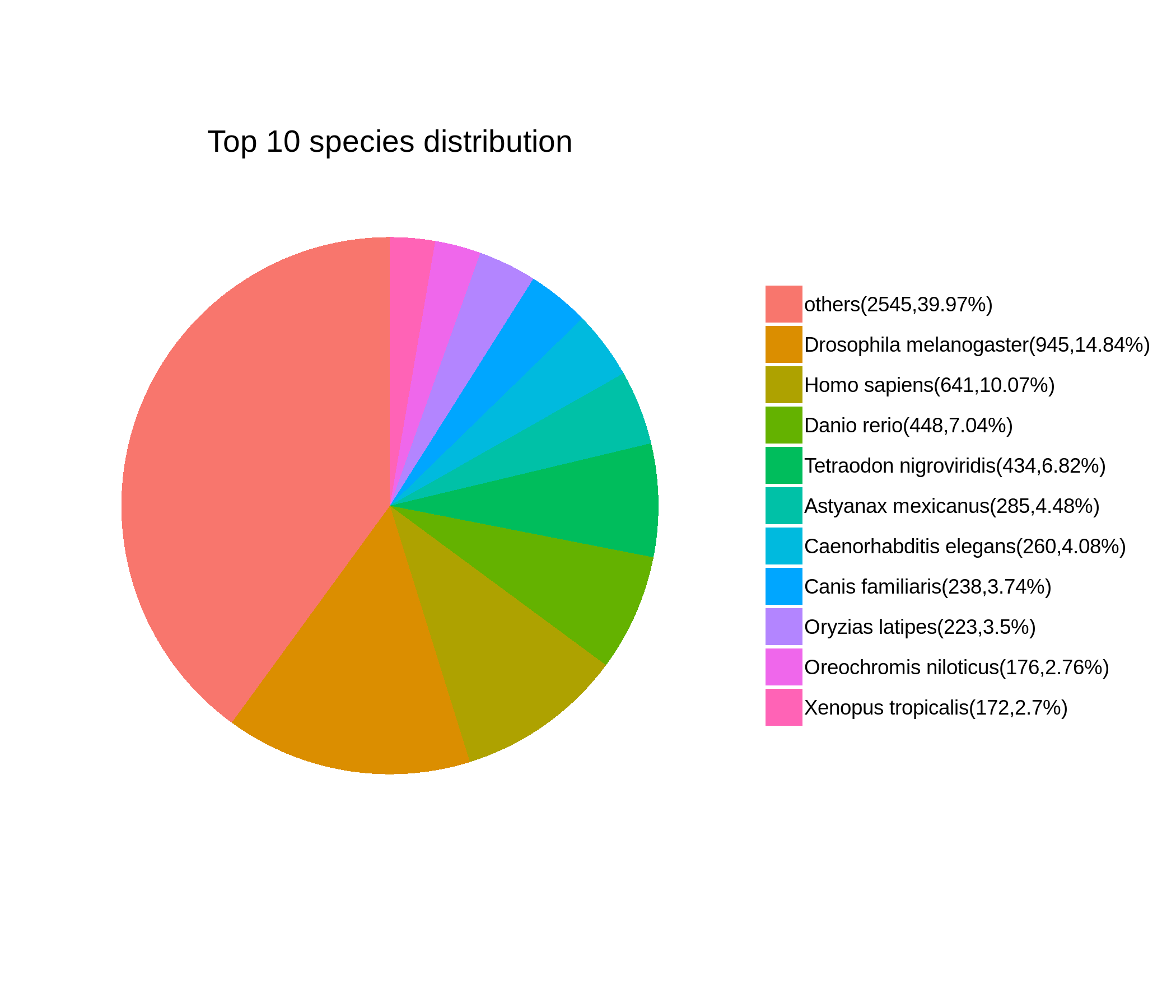

Supplement: Supplementary file 1 [file biomolecules-13-00460-s001.zip › Figure S3 Unigene.TFs.species.png]

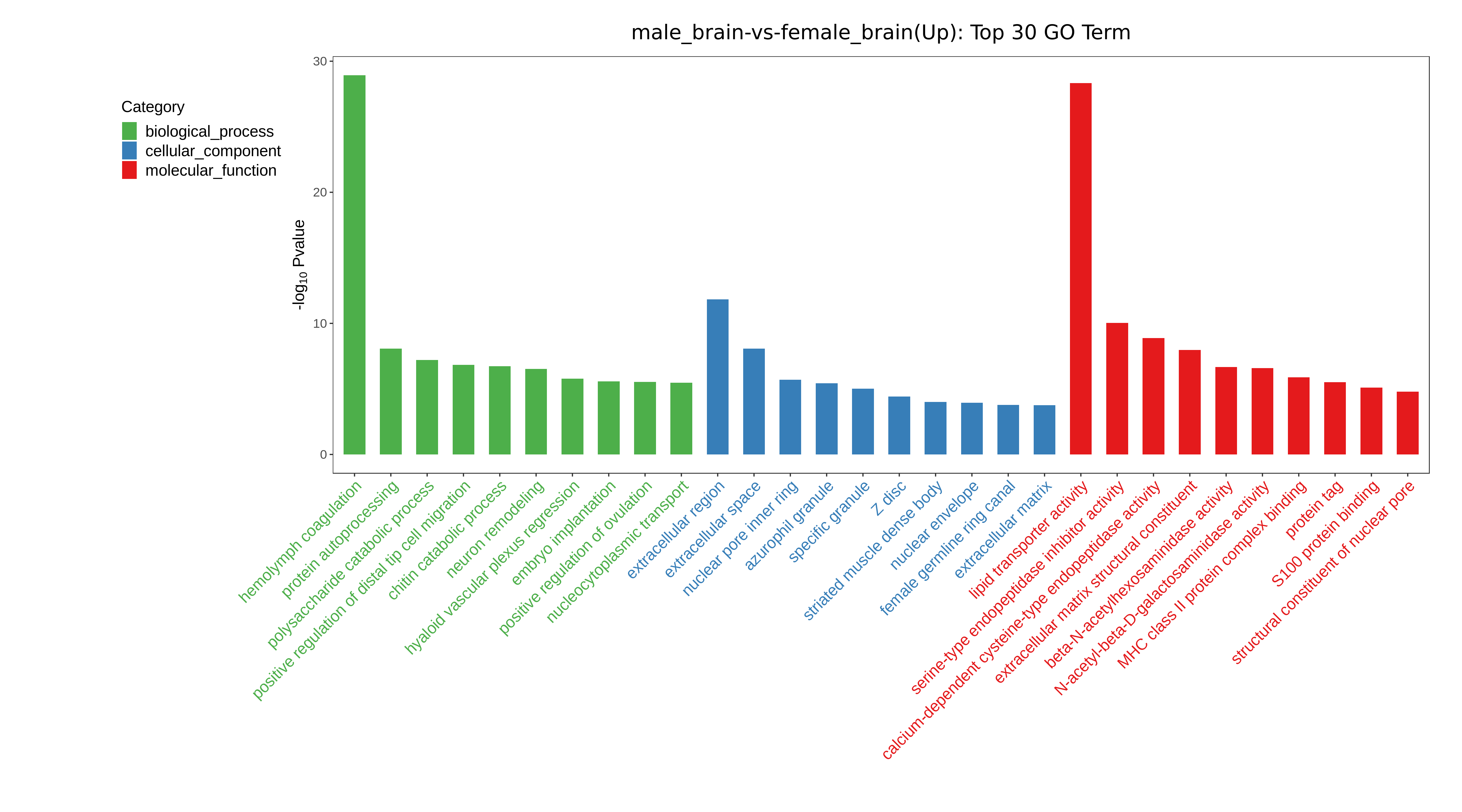

Supplement: Supplementary file 1 [file biomolecules-13-00460-s001.zip › Figure S4 brain GO.top.Up.png]

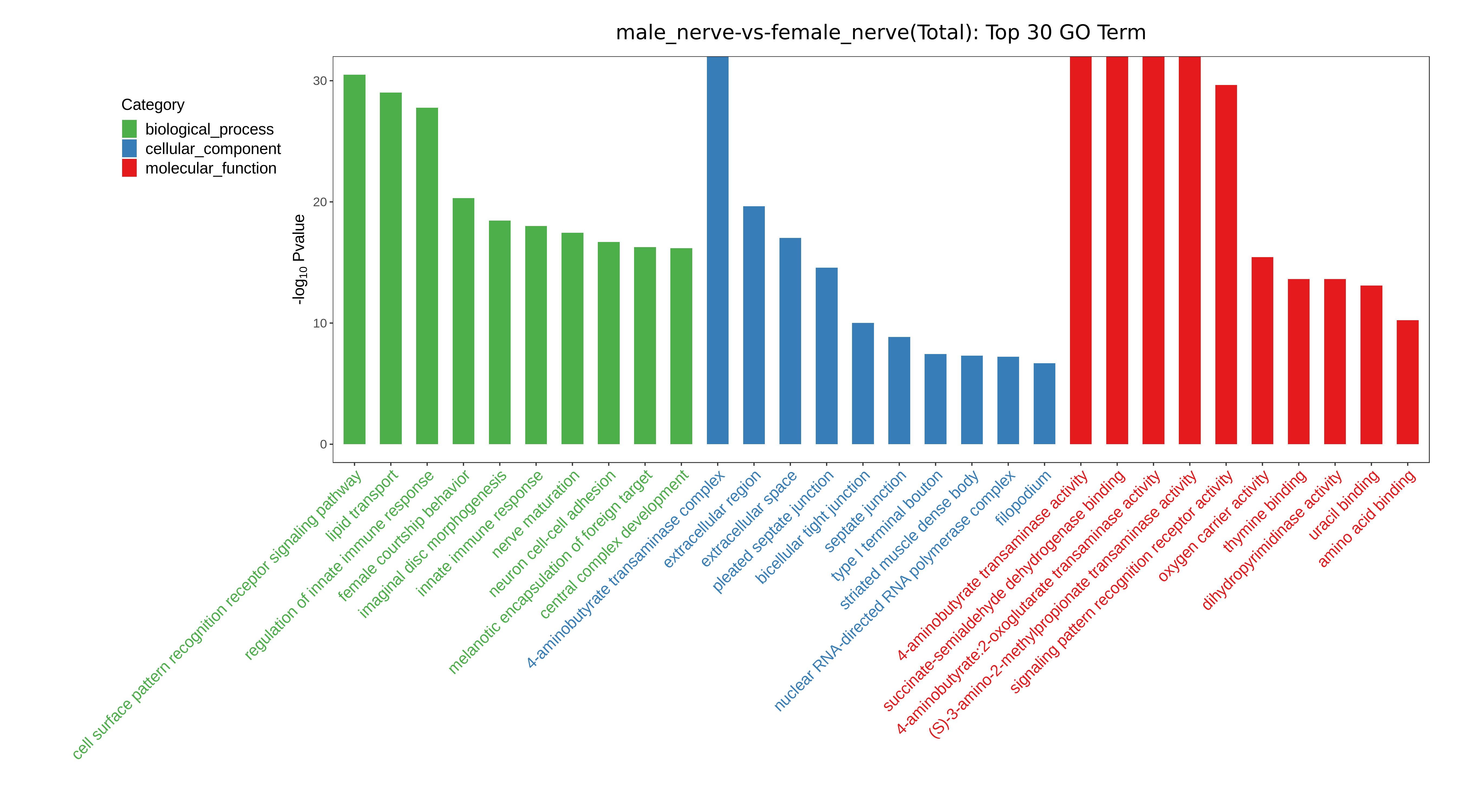

Supplement: Supplementary file 1 [file biomolecules-13-00460-s001.zip › Figure S5 nerve GO.top.Total.png]

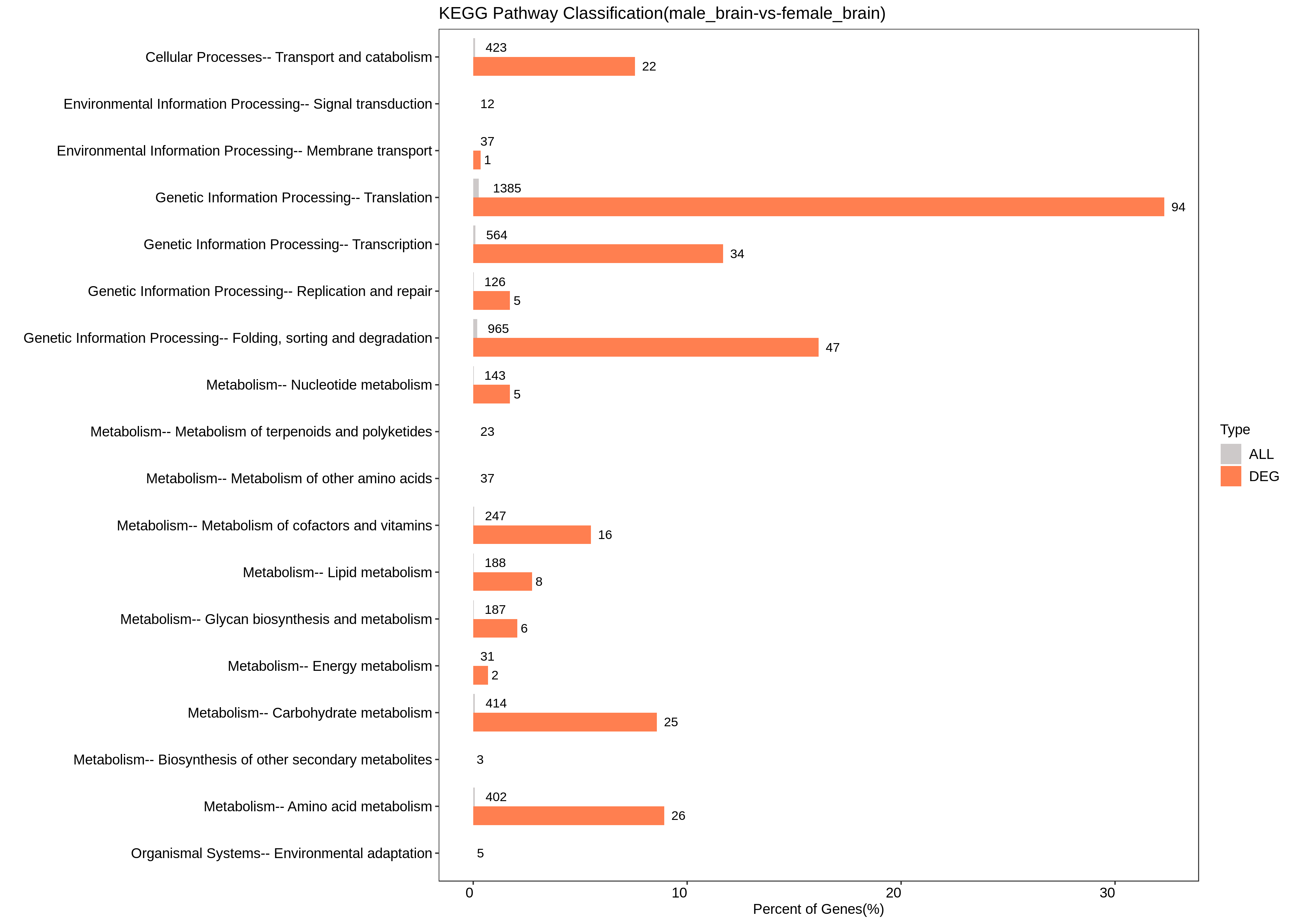

Supplement: Supplementary file 1 [file biomolecules-13-00460-s001.zip › Figure S6 brain ALL_vs_DEG.KEGG_Classification.png]

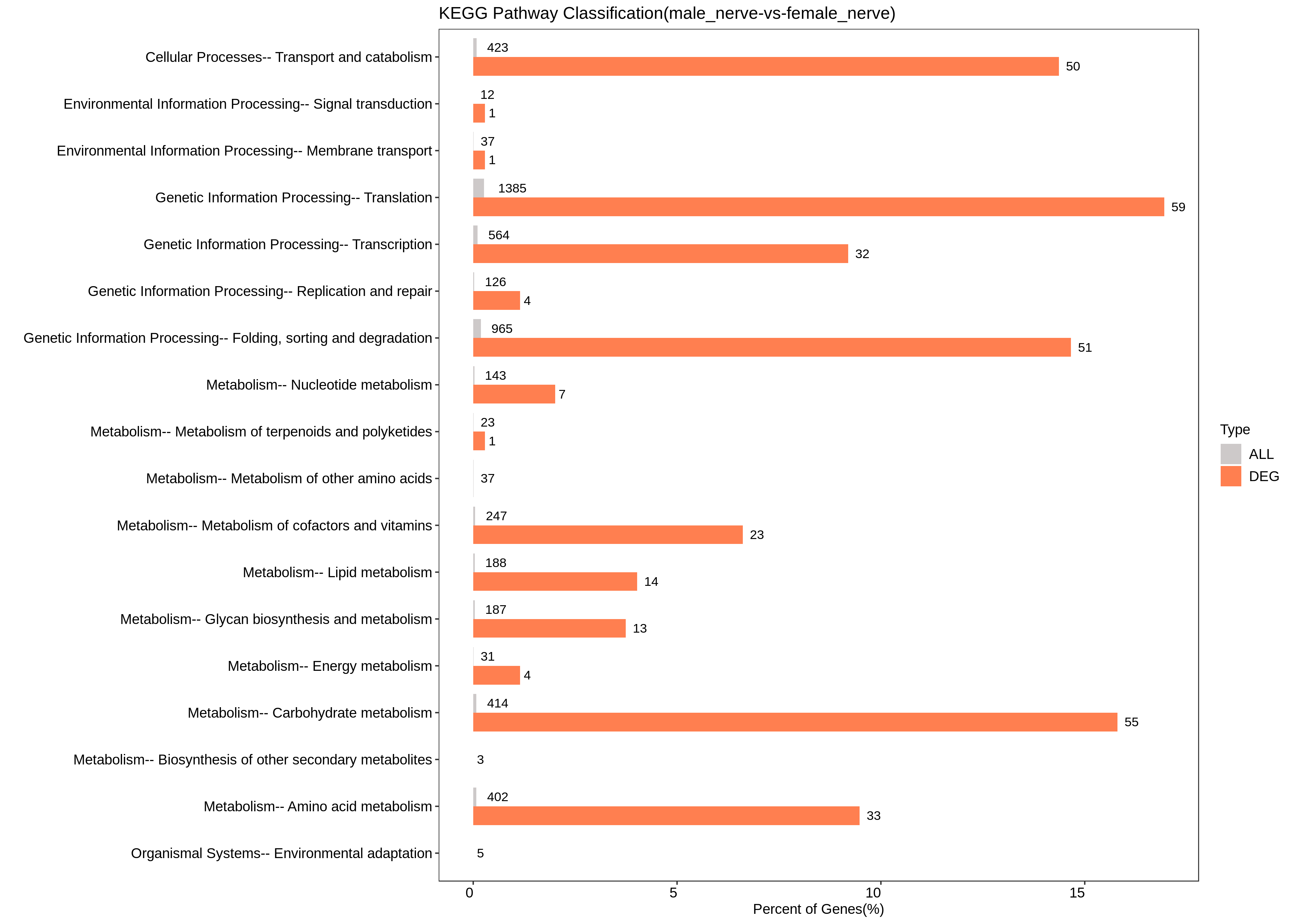

Supplement: Supplementary file 1 [file biomolecules-13-00460-s001.zip › Figure S7 nerve ALL_vs_DEG.KEGG_Classification.png]
